# Influence of hepatitis B virus co-infection on virological and immunological response to antiretroviral treatment among HIV patients attending comprehensive care clinics in Makueni County, Kenya

**DOI:** 10.11604/pamj.2021.38.103.25793

**Published:** 2021-02-01

**Authors:** Geoffrey Mutisya Maitha, Gideon Kikuvi, Peter Wanzala, Fredrick Kirui

**Affiliations:** 1 School of Public Health, Jomo Kenyatta University of Science Agriculture and Technology, Nairobi, Kenya,; 2 Kenya Medical Research Institute, Nairobi, Kenya

**Keywords:** HIV, hepatitis B virus, CD4 count, viral load, antiretroviral

## Abstract

**Introduction:**

the effect of hepatitis B virus (HBV) infection on the natural history of human immunodeficiency virus (HIV) disease remains uncertain. Therefore, this study was conducted to determine the association of HBV co-infections with CD4 count and viral load levels in response to antiretroviral treatment among HIV patients attending comprehensive care clinics in Makueni County (Kenya).

**Methods:**

this was a prospective case-control study among patients seeking HIV services in three hospitals of Makueni County (Kenya). Newly diagnosed HIV mono-infected patients (controls) and HIV/HBV co-infected (cases), 18 years and above who had not started antiretrovirals (ARVs) participated. A total of 258 patients gave informed consent and participated. HIV mono-infected (controls) produced 129 while HIV/HBV (cases) gave 129 participants. P-values ≤ 0.05 were considered significant.

**Results:**

the majority (164, 63%) of the study participants were females for both arms of the study. The mean age of the participants was 31±0.402 years and majority of them were between the age of 26-30years old. At the beginning and end of the study the mean viral load for HIV/HBV co-infected individuals was (30169 and 1731) copies/ml while that of CD4 count was (327 and 459) cells/ul, and that of HIV mono-infected was (21860 and 1689) copies/ml and CD4 count of (421 and 437) cells/ul respectively. After enrolling them into antiretroviral therapy (ART) treatment and after six months of follow-up there was significant drop in viral load and significant increase in CD4 count for both groups at p<0.001 using logistic regression.

**Conclusion:**

HIV patients co-infected with hepatitis B virus had high viral load and low CD4 count compared to HIV monoinfected patients however with introduction of ARVs there was improvement in both groups with the highest noticed among the HIV/HBV co-infected patients.

## Introduction

The Hepatitis B Virus (HBV) and Human Immunodeficiency Virus (HIV) are viruses that share certain epidemiological characteristics such as risk populations and transmission routes. This puts HIV positive individuals at risk of co-infection with hepatitis B [1-3]. Elimination of viral hepatitis as a major public health threat by 2030 is part of the Sustainable Development Goals (SDGs). The World Health Organization (WHO) adopted a global strategy in 2016, which aims to reduce the number of new cases of chronic hepatitis B and C by 90% and the number of deaths associated with these diseases by 65% by 2030 [4]. Testing, treatment with ARVs, and virologic suppression are crucial elements of this strategy. Hepatitis B is a major concern in Africa, especially among HIV-infected patients who have a greater risk of liver failure, cirrhosis, hepatocellular carcinoma, and death [5]. Of the 36.7 million people living with HIV worldwide in 2015, approximately 2.7 million (7.4%) were coinfected with hepatitis B virus (HBV) [6]. Nearly three quarters of the latter resided in Africa. In a recent meta-analysis, the prevalence of HBV infection in people living with HIV was estimated at 8.4% worldwide and up to 12.4% in West and Central Africa [5].

Few studies in Kenya have been done on HIV/HBV co-infection in relation to CD4 count and viral load. The objective of this study was to determine the association of HBV co-infections with CD4 count and viral load levels in response to antiretroviral treatment among HIV patients attending comprehensive care clinics in Makueni County (Kenya). Results obtained from this study will inform policy formulation towards HIV care, prevention and treatment programs and help to improve on care and treatment of patients with HBV co-infections.

## Methods

**Study site:** the study was carried out in the year 2017/2018 in three selected comprehensive care clinics in Makueni County which is 144 KM south of Nairobi the capital city of Kenya with a population of 930,530 with a current prevalence of HIV of 5.6%. The three facilities were Makindu, Wote and Emali hospital, where high number of HIV patients seek comprehensive care services within the county.

**Study design and study populations:** this was a prospective case-control study among HIV mono-infected (controls) and HIV/HBV co-infected (cases) patients. The study looked at the difference in CD4 count and viral load between the two groups at baseline (enrollment) and follow-up which was conducted once at six months to ascertain if there is any association with HBV co-infection. At enrollment both groups were initiated on ARVs and their CD4 count and viral load taken whereby these tests were also done during follow-up.

**Inclusion criteria for HIV mono-infected (controls) patients:** should be 18 years and above, newly diagnosed for HIV and not on ARVs, not vaccinated for hepatitis B before and has given informed consent to participate in the study.

**Inclusion criteria for HIV/HBV co-infected (cases) patients:** should be 18 years and above, newly diagnosed for HIV/HBV co-infection and was not on ARVs at the time of enrollment and has agreed to give informed consent to participate in the study.

**Sample size determination:** the study utilized sample size formula for comparing two proportions by Casagrande *et al*. (1978) to obtain the minimum sample size. The minimum sample size per group was calculated as 107. Allowing for 20% non- completeness/loss to follow up; the sample size was adjusted upwards to 129. A total of 258 participants gave informed consent and were recruited for the study.

$$N=\frac{{\left\{Z_{\alpha }\sqrt{2\bar{pq}}+Z_{\beta }\sqrt{p_{1}\left[1+R-p_{1}\left(1+R^{2}\right)\right]}\right\}}^{2}}{{\left\{p_{1}\left(1-R\right)\right\}}^{2}}$$

Where; N is the sample size for each group

$$\bar{p}=1/2p_{1}\left(1+R\right),\bar{q}=1-\bar{p}$$

**P**_1_=is the anticipated incidence of the factor of interest (outcome) among the unexposed (expressed as a proportion), i.e. 0.55

**P**_2_=is the anticipated incidence of the factor of interest (outcome) among the exposed (expressed as a proportion), i.e. 0.75

**R**= is the anticipated relative risk of having the factor of interest (outcome) (R= P_2_/ P_1_) 0.75/0.55 = 1.36

Z_α_= is the standard normal deviate for a given level of significance (1.962 for 5% level of significance)

Z_β_= is the standard normal deviate for a given power (0.84 for a power of 80%)

p^-^=1/2 {0.55(1+136)}=0.65; q^-^=1-0.65=0.35

N=({1.96v2*0.65*0.35+0.84v(0.55[1+1.36-0.55(1+[(1.36)]^2 )}^2))/({0.55 (1-0.65)}^2 )=107.2

***NOTE:*** *exposure variable = hepatitis B*

**Sampling procedure:** Makueni County has 57 comprehensive care clinics and three facilities were purposively selected for the study because they have high number of HIV patients seeking services and also represent rural and urban areas. Makueni county referral has approximately monthly new HIV enrollments of 60 patients, Makindu hospital has 50 and Emali clinic has 35. To get the number of participants to be produced in each arm (control and cases) we used proportionate method guided by the monthly data from each facility. After testing the participants those with HIV only were allocated to the control group while those who tested for both HIV/HBV were allocated to cases. Equal numbers were allocated to controls and cases. Fifty three, forty five and thirty one patients for each of the two arms were recruited from Makueni county referral, Makindu hospital and Emali clinic respectively. Consecutive sampling was used to recruit the study participants from each facility.

**Collection of data on socio-demographic, socio-economic characteristics and laboratory:** data on socio-demographic and socio-economic characteristics was collected using questionnaires with help of a health care provider recruited and trained to be part of the research team. Laboratory data on CD4 count and viral load levels was also collected for both control and cases for comparisons in changes at baseline and follow-up.

**Determination of HIV and HBV status, CD4 and viral load:** hepatitis B screening for each participant and CD4 count was done at the same facility where the study was taking place and laboratory results entered to participants’ questionnaire the same day while viral load results were later entered to participants’ database after receiving them from KEMRI. Both HIV and HBV was conducted using rapid test methods. Participants in the non- exposed group (negative for HBV) were vaccinated for hepatitis B after enrolment to the study. In the two groups baseline data on CD4 count and viral load were collected at enrolment to the study before starting them on ARVS. Both groups were initiated on ARVS and followed up for six months. Data on CD4 count and viral load were collected at the end of six month follow-up period. Approximately, 2mls of blood was collected using EDTA vacutainer bottle for every study participant at baseline and at the end of the study (sixth month of follow up). Blood was stored at room temperature and analysis for CD4 was done using Partec Cyflow machine while HBV was detected from serum using an advanced quality one-step rapid test kit at the facility since all these facilities have laboratories which can carry out these tests. The dried blood spot (DBS) samples for viral load were collected and kept at room temperature and taken to the nearby G4S (courier) office for transportation to KEMRI (Centre for virus Research) Nairobi for processing. Indivinduals with less than 1000 copies/ul were considered to have achieved viral load suppression

**Data management and analysis:** double data entry method was used to ensure quality and consistence. Data cleaning was undertaken to identify errors made during questionnaire filling and data entry. Data processing and analysis was done using the Statistics Package for Social Science (SPSS) software version 20.0. The quantitative data was analysed using SPSS software. Descriptive data was presented using frequency tables. Cross tabulation involving Chi square test was used to compare the relationship between variables (CD4 count and viral load). Multivariate analyses using logistic regression analysis to test the null hypothesis of the study. P-values ≤ 0.05 were considered significant.

**Ethical considerations:** the scientific and ethical research committee of Kenya Medical Research Institute granted ethical approval to carry out the study (SERU/RES/7/3/1). Permission was sought from leaders in the County Ministry of Health department of HIV/AIDS and Medical superintendent of the facilities where the study was taking place. The participants´ informed consent was sought and codes were used instead of real names of participants.

## Results

**Socio-demographic and socio-economic characteristics of the study participants:** majority (63%) of the study participants in both HIV/HBV positive and HIV/HBV negative arms of the study were females. The mean age of the participants was 31±0.402 years and most (32.8%) of them in the HIV/HBV cohort were aged 26-30 years while in HIV cohort most (26.2%) were aged 31-35 years. More than seventy eight percent of the participants in each of the groups were married. Of these 77% in the HIV/HBV and 98% in the HIV group were in monogamous marriage. More than half of the study participants in each of the groups had attained secondary level of education. In both groups more than a third of the study participants were in formal employment and majority (>50%) lived in rural areas. There were no significant differences (p> 0.05) in the characteristics of the participants in the two groups (Table 1).

**Table 1:** socio-demographic and socio-economic characteristics of the study participants

Demographic Information	Characteristics	Hepatitis B virus and HIV status			
		HBV/ HIV (Cases) (n) (%)	HIV (Control) (n) (%)	Total (n) (%)	P-value
Gender	Male	47(36.7)	47(36.2)	94(36.4)	0.925
	Female	82(63.3)	82(63.8)	164(63.6)	
	Total	129(100.0)	129(100.0)	258(100.0)	
Age category	21-25	28(21.9)	24(19.2)	52(20.5)	0.560
	26-30	42(32.8)	33(25.4)	75(29.1)	
	31-35	30(23.4)	34(26.2)	64(24.8)	
	36-40	18(14.1)	26(20.0)	44(17.1)	
	41-45	6(4.7)	9(6.9)	15(5.8)	
	46-50	3(2.3)	1(0.8)	4(1.6)	
	51-55	2(0.8)	2(1.5)	4(1.2)	
	Total	129(100.0)	129(100.0)	258(100.0)	
Marital status	Married	103(79.7)	102(78.5)	205(79.1)	0.714
	Single	19(14.8)	22(17.7)	41(16.3)	
	Divorced	4(3.1)	4(3.1)	8(3.1)	
	Windowed	3(2.3)	1(0.8)	4(1.6)	
	Total	129(100.0)	129(100.0)	258(100.0)	
Education level	Primary	28(21.9)	29(22.3)	57(22.1)	0.353
	Secondary	69(53.1)	68(52.3)	137(52.7)	
	Tertiary (college/University)	29(22.7)	32(25.4)	61(24.0)	
	No formal education	3(2.3)	0(0.0)	3(1.2)	
	Total	129(100.0)	129(100.0)	258(100.0)	
Employment status	Self employed	45(35.2)	46(35.4)	91(35.3)	0.865
	Employed	48(36.7)	50(39.2)	98(38.0)	
	Not employed	36(28.1)	33(25.4)	69(26.7)	
	Total	129(100.0)	129(100.0)	258(100.0)	
Residence	Urban area	63(49.2)	51(40.0)	114(44.6)	0.136
	Rural area	66(50.8)	78(60.0)	144(55.4)	
	Total	129(100.0)	129(100.0)	258(100.0)	

The p-value compares relationship between demographic information in relation to HIV and HBV status among study participants.

**HIV viral load levels among HIV/HBV co-infected and HIV participants at baseline and six month follow up:** at baseline none of the participants with HBV co-infection had undetectable viral load status and only two participants among the HIV mono-infected group had undetectable levels. Viral load status did not show statistical significance at baseline (P=0.342). After introduction of ARVs to both groups and at six months follow up there was great improvement for both HIV/HBV co-infected and HIV mono-infected. Highest improvement on viral load status was observed among HIV/HBV co-infected clients however this did not have any statistical significance at p=0.292 (Table 2).

**Table 2:** significance difference of viral load status a mong HIV/HBV co-infected and HIV participants at baseline and follow up

	Current status of viral load results	HIV/HBV (cases) (n) (%)	HIV (controls) (n) (%)	Total (n) (%)	P-value	Kappa
**Baseline**	Viral load suppressed (50-999)	42(48.2)	44(51.8)	86(100)	0.342	-0.001
	Un detectable levels (Less than 50)	0(0)	2(100)	2(100)		
	High (more 1000)	87(50.9)	83(49.1)	170(100)		
**Follow up**	Viral load suppressed (50-999)	60(49.2)	62(50.8)	122(100)	0.292	-0.037
	Un detectable levels (less than 50)	38(57.6)	28(42.4)	66(100)		
	High (more 1000)	31(44.3)	39(55.7)	70(100)		

The p-value compares the relationship between patients viral status in relation to hepatitis B virus co-infection while the kappa statistics shows the level of agreement between the researchers.

**Figure 1:**
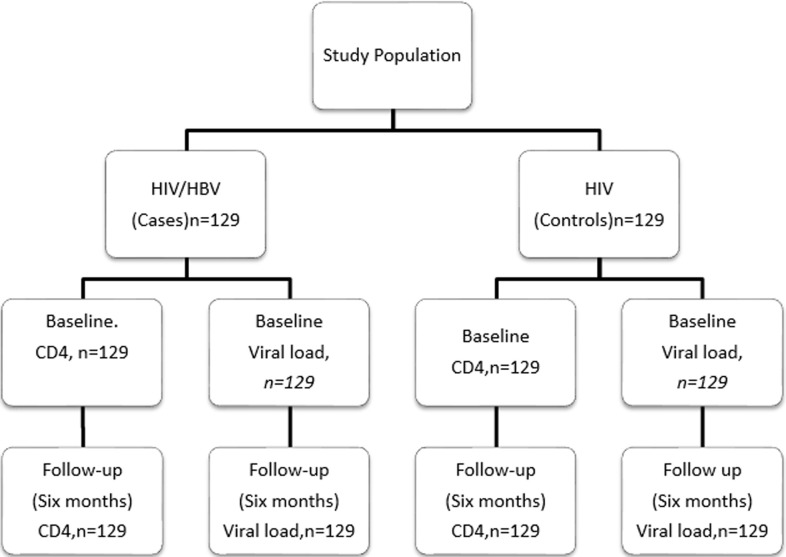
progression of HIV/HBV and HIV participants at baseline and follow-up

**Association between hepatitis B virus co-infection and virological and immunological response to antiretroviral treatment among HIV patients attending comprehensive care clinics in Makueni County (Kenya):** at baseline the mean viral loads were 30169±103693 copies/ml and 21860±77690 copies/ml in the HIV/HBV co-infected and HIV mono-infected patients respectively. During follow up at sixth month and introduction of ARVs both groups of patients showed reduced viral load levels which were statistically significant at (p<0.001) highest change in viral load levels was noticed among the HIV/HBV co-infected patients. Similar tred was observed for CD4 count among the groups where there was statitistical significance between baseline and follow up at p<0.001. The mean CD4 count increased in the two groups however highest improvement was noticed among the HIV/HBV co-infected indivinduals compared to those with only HIV infection. The mean CD4 count change from base line and follow up for HIV/HBV co-infected was 132 cells/ul while that of HIV mono-infected was 16 cells/ul after ART initiation as shown in (Table 3) (Figure 1).

**Table 3:** association between hepatitis B virus co-infection and virological and immunological response to antiretroviral treatment among study participants

Laboratory test	Survey	Status	N	Mean	Std. deviation	F	P-value	Partial eta squared
**Viral load (copies/ ml)**	Baseline	HIV/HBV (cases)	129	30169.69	103692.763	18.054	0.000	0.034
		HIV(control)	129	21860.25	77690.345			
	Follow up	HIV/HBV (cases)	129	1731.40	8286.449			
		HIV(control)	129	1689.23	5118.269			
**CD4 count (cells/ ul)**	Baseline	HIV/HBV (cases)	129	327.66	136.825	43.756	0.000	0.079
		HIV(control)	129	421.88	121.898			
	Follow up	HIV/HBV (cases)	129	459.26	127.342			
		HIV(control)	129	437.23	117.804			

The p-value compares changes in viral load and CD4 count at baseline and follow-up and therefore determining if the change is significant between the two groups while F-test is comparing the mean of the variables at baseline and follow-up between the cases and controls.

## Discussion

This study has shown that CD4 count for the HIV mono-infected patients was significantly higher than that of the patients co-infected by HBV. This is in line with findings from a hospital based descriptive cross sectional study conducted in Nigeria [7] to determine the effect of hepatitis B virus co-infection on CD4 cell count and liver function of HIV infected patients, study by [8] reported that CD4 count was non-significantly lower in co-infected patients.

Study by Obeagu [9] on impact of HIV and hepatitis B Virus reported contrary results. They noted significant decrease (P<0.05) in CD4+ T cells of the HIV/HBV co infected subjects compared to HIV mono-infected subjects.This shows that HBV may act as an accelerator of pathogenesis of HIV. It has a suppressive effect on the CD4+ T cells as seen in the HIV/HBV co infection. This increases chances of morbidity and mortality rate of those infected if not well treated immediately. Those infected with HIV must avoid being infected with HBV to avert the debilitating danger to the immune system and haematologic system too. This could be due to different treatment guideline policies. Their patients were also not initiated on ARVs during the time of the study and this could cause significant decrease in CD4 count among the co-infected, at baseline when we were recruiting our study participants the HIV/HBV co-infected had low mean CD4 count than the mono-infected but after initiation of ARVs there was significant increase in CD4 count among the co-infected. This explains why there was significant increase of CD4 count among this group in this particular study as ARVs are known to improve immune system of HIV infected patients.

Our study revealed reduced viral load at six months and significant improvement was noticed to HIV/HBV co-infected patients compared to those with HIV only. At six months some patients had achieved HIV viral suppression. This was associated with ART initiation of naive patients during recruitment to participate in the study and good drug adherence. ARVs are known to reduce or suppress the virus and with good adherence the patient is likely to achieve viral load suppression [10]. Study on antiretroviral adherence and virological outcomes in HIV-positive patients in KwaZulu-Natal province found inconsistent results from our study where a statistically significant relationship between adherence and viral suppression was not demonstrated this could be due to the method they were using to assess drug adherence to their participants as they used pharmacy refill method he suggested pharmacy refill records cannot be recommended as an alternative method of monitoring response to antiretroviral therapy, but laboratory tests including CD4 cell count and or viral load must be combined with the pharmacy refill method for monitoring of antiretroviral therapy in HIV-positive patients, ARVs tend to reduce viral load and increase CD4 count among HIV patients.

Participants who were taking alcohol or smoking had high mean viral load compared to those who didn´t take. This may be due to poor adherence since most of people who take alcohol may forget to take their medication or take it at late hours, others may have poor feeding habit despite their good adherence affecting nutritional issues and functioning of ART within the system [11]. Effect of alcohol on CD4+ cell decline appears to be independent of ART, through a direct action on CD4 cells, although alcohol and substance abuse may lead to unmeasured behaviors that promote HIV disease progression. The effect of alcohol abuse on viral load, however, appears to be through reduced adherence to ART. In his main findings showed that frequent alcohol consumption is a predictor of CD4+ cell decline, as evidenced by a significantly greater decline of CD4 cell counts in participants who used ART, as well as those who were ART naive. Moreover, the combination of frequent alcohol and crack-cocaine use also significantly decreased CD4+ cell count over time, and the decrease appears to be independent of ART. In addition, frequent alcohol use increased plasma HIV viral load, although this relationship was statistically significant only in participants who were on ART. Thus, frequent alcohol consumption appears to affect HIV disease progression by accelerating the decline of CD4+ cell count and increasing viral load only in those receiving ART [11,12] study on Alcohol consumption patterns and HIV viral suppression among persons receiving HIV care in Florida found similar results with our current study where exceeding weekly recommended levels of alcohol consumption (heavy drinking) was significantly associated with poor HIV viral suppression and ART non-adherence, while binge drinking was associated with suboptimal ART adherence in this sample. He recommended clinicians should attempt to address heavy drinking in their patients with HIV as part of counselling.

Study conducted in Kisumu district hospital [13] found that the mean CD4 cell count for patients with co-infection was lower, (120 (+/-112) cells/mm^3^) than for patients with HBV mono-infection, 694 (+/-140) cells/mm^3^ which is in agreement with our current study. A similar study on sero-prevalence of HBV and associated risk factors among HIV positive individuals attending ART clinic at Mikelle hospital, Tigray, Northern Ethiopia, which found that HIV/AIDS positive individuals with reduced CD4 count, <200 cells/μl, showed a significant association with HBsAg seropositivity at p=0.05 [14]. Similar results were also reported [13] where he found that the mean CD4 count of HIV mono-infected patients to be significantly higher than that of co-infected patients at p=0.014 this is in agreement with our current study. However, the limitation on this study was that the participants selected to participate were HIV/HBV co-infected who presented with jaundice only.

## Conclusion

The study has shown that CD4 count is high among HIV mono infected patients as compared to HIV patients co-infected with hepatitis B virus while viral load levels are high among HIV co-infected compared HIV mono infected patients however with introduction of ARVs the CD4 count improves(increases) for the two groups while the viral load levels reduces.

### What is known about this topic

- HIV/HBV co-infection may cause liver cirrhosis or hepatotoxicity with introduction of ARVs. This causes elevated levels of liver enzymes when diagnosed.

### What this study adds

- Both HIV/HBV co-infected and HIV mono-infected indivinduals show improved viral load (decreased) and CD4 count (increased) after starting ARVs however greatest improvement is noticed among HIV/HBV co-infected indivinduals.
